# BMI and Varus Malalignment Compound to Define a High-Risk Phenotype for Compartment-Specific Knee Osteoarthritis Progression

**DOI:** 10.64898/2026.04.15.26350819

**Published:** 2026-04-17

**Authors:** McKenzie S. White, Feliks Kogan, Scott L. Delp, Constance R. Chu, Seth L. Sherman, Anoosha Pai S, Garry E. Gold, Akshay S. Chaudhari, Anthony A. Gatti

**Affiliations:** 1. Department of Radiology, Stanford University, Stanford, CA, USA; 2. Department of Bioengineering, Stanford University, Stanford, CA, USA; 3. Department of Orthopedic Surgery, Stanford University, Stanford, CA, USA; 4. Department of Biomedical Data Science, Stanford University, Stanford, CA, USA

**Keywords:** Obesity, Risk Stratification, Tibiofemoral Joint, Joint Loading, Hip-Knee-Ankle Angle

## Abstract

**Objectives:**

Knee osteoarthritis (KOA) is a leading cause of disability, yet which patients will experience structural decline remains unclear. Body mass index (BMI) and lower limb alignment are established risk factors for KOA, but their independent and interactive effects on compartment-specific cartilage loss and total knee replacement (TKR) have not been characterized at scale.

**Methods:**

We analyzed 5,832 limbs from 3,016 participants in the Osteoarthritis Initiative followed over 7 years. Cartilage thickness in the weight-bearing medial and lateral femur and tibia was quantified, and lower limb alignment was measured using hip-knee-ankle (HKA) angle obtained from full-limb radiographs. Linear mixed-effects models estimated the independent and interactive effects of BMI and lower limb alignment on longitudinal cartilage thinning, and mixed-effects logistic regression modeled TKR risk.

**Results:**

In the medial compartment, BMI and varus alignment interacted multiplicatively, with their combined effect exceeding the sum of independent contributions (femur: p = 0.011; tibia: p< 0.001). At +10kg/m^2^ BMI and +10° varus, the rate of medial femur cartilage thinning was 243.5% faster than the reference rate. In the lateral compartment, BMI and valgus alignment were independently associated with faster cartilage thinning, with no significant interaction. TKR risk increased exponentially with HKA deviation (odds ratio [OR] = 1.38 per 1°; ~five-fold at 5° malalignment) but was not associated with BMI.

**Conclusion:**

BMI and lower limb alignment influence structural KOA progression through compartment-specific pathways. The multiplicative interaction in the medial compartment identifies high BMI combined with varus malalignment as a discrete high-risk phenotype, with implications for clinical risk stratification and disease-modifying intervention design.

## INTRODUCTION

Knee osteoarthritis (KOA) is a leading cause of pain and disability worldwide,[1] yet its progression is highly heterogeneous.[2] Identifying patients at greatest structural risk, those most likely to experience rapid cartilage loss and progress to total knee replacement (TKR), remains a critical challenge for clinical management and disease-modifying trial design.[3, 4] Two of the most readily measured clinical characteristics, body mass index (BMI) and lower limb alignment, are established independent risk factors for KOA,[5–9] but their direct link to structural progression by compartment has not been established at scale.

KOA is widely recognized as a mechanically driven disease[10]. During gait, BMI and lower limb alignment influence joint loading through distinct pathways: body mass scales the ground reaction force and thus total knee joint load, while varus-valgus alignment alters how that load is distributed between the medial and lateral compartments. Consistent with this mechanical framework, both factors are independently associated with structural disease. Individuals classified as overweight have a substantially higher odds of KOA incidence (odds ratio [OR] = 3.3)[5] and progression (relative risk = 2.4).[6] Malalignment has been associated with up to a five-fold increase in the odds of compartment-specific KOA progression.[6–9] If progression is driven by the total load experienced in a given compartment, BMI and alignment should statistically interact, thereby producing a disproportionately greater structural degeneration in individuals with both higher BMI and greater degrees of malalignment.

However, whether BMI and lower limb alignment interact to influence compartment-specific KOA progression remains unclear. Prior studies have produced conflicting results: some report independent effects,[6, 11] others suggest BMI-related progression is most evident at intermediate levels of malalignment or is mediated by varus malalignment,[11, 12] and still others find BMI-related progression only in neutral or valgus aligned knees.[13] These inconsistencies likely reflect shared methodological limitations. Most prior work has relied on joint space narrowing (JSN) as an indirect measure of cartilage loss, rather than directly measuring cartilage thickness that more sensitively and directly measures structural decline[14–16]. Additionally, lower limb alignment has often been estimated from the femorotibial angle (FTA) on fixed-flexion knee radiographs, which correlates only moderately with full-limb hip-knee-ankle (HKA) measurements (r = 0.68-0.72).[6, 11, 17] Finally, sample sizes have generally been too small to reliably detect interaction effects across the spectrum of BMI and lower limb alignment.

Historically, the labor-intensive nature of quantitative cartilage assessment has impeded large-scale characterization of longitudinal cartilage thickness changes. Deep learning-based segmentation, combined with imaging resources such as the Osteoarthritis Initiative (OAI), now enable longitudinal assessments of cartilage thickness at unprecedented scale. In parallel, full-limb radiographs available in the OAI provide accurate and reproducible measures of lower limb alignment across thousands of participants. Together, these resources create a unique opportunity to quantify how BMI and lower limb alignment independently and jointly influence KOA progression by probing cartilage structure directly.

Accordingly, the objective of this study was to quantify the independent and interactive effects of BMI and lower limb alignment on rates of compartment-specific cartilage thinning and the risk of TKR using large-scale quantitative MRI and full-limb HKA radiographic data from the OAI. By directly linking these readily measured clinical factors to longitudinal cartilage structural decline and TKR risk, we aim to inform clinical risk stratification and identify patient phenotypes in whom excess BMI, malalignment, or their combination confer the greatest risk of structural progression.

## METHODS

### Dataset

Data were derived from the OAI, a publicly available, longitudinal cohort study designed to identify biomarkers and risk factors for KOA. Participants were included in our analysis if they met two eligibility criteria. First, they were required to have a full-limb radiograph with a measured HKA angle and complete demographic information (sex, age, BMI, and Kellgren-Lawrence [KL] grade) available at the same visit, which served as the baseline visit of our study. Second, each limb needed to have at least two knee MRI examinations obtained at or after the baseline visit.

Lower limb alignment was measured by two independent central reading centers through OAI Projects 32 and 60, which provided semi-automatic HKA angle measurements from full-limb radiographs. Project 60 has demonstrated excellent agreement with manual measurements (intraclass correlation coefficients [ICCs] = 0.977-0.999) and strong inter- and intra-reader reliability (ICCs = 0.839-0.998). Although comparable reliability metrics for Project 32 are not published, its HKA measurements show high agreement with Project 60 (ICC = 0.98; mean difference of 0.1°). between the two sources, we utilized the average of these reported HKA angle measurements as the reference HKA angle for this study. In cases where measurements were provided by only one project, that single value was used.

For all included limbs, we extracted Double Echo in Steady State (DESS) MRI scans from the HKA-measurement visit (baseline) and all subsequent visits. Femoral and tibial bone and cartilage were segmented using validated deep-learning based methods (tibiofemoral cartilage Dice similarity coefficients = 0.876-0.907, average symmetric surface distance = 0.13-0.17mm)[18] and corresponding surface meshes were generated for each structure.[19] To ensure consistent regions of interest across all participants and to account for full cartilage thickness lesions (0 mm thickness), an average reference mesh was generated for the femur and tibia using a neural shape model previously trained on 6,325 knees from the broader OAI cohort.[20] Standardized regions of interest were defined using established standards[21] and included vertices where ≥95% of the healthy cohort had overlying cartilage. Cartilage thickness was computed for each point on the bone surface by projecting a normal vector and calculating the Euclidean distance between its intersections with the bone-cartilage interface and the articular surface. Using this approach, we extracted mean cartilage thickness in four sub-regions: weight-bearing medial and lateral femur, and medial and lateral tibia. Previous analyses demonstrated that these methods have strong agreement with manual carolage thickness measurements in 596 knees from the Foundaoon for the Naoonal Insotutes of Health (FNIH) OAI cohort (medial femur: r = 0.926, lateral femur: r = 0.910, medial obia: r = 0.914, lateral obia: r = 0.943) and have greater sensiovity to longitudinal change than manual measurements: standardized response means were larger for automated measurements across compartments (medial femur: −0.51 [−0.58, −0.44] vs. −0.46 [−0.52 to −0.39]; lateral femur: −0.18 [−0.26, −0.11] vs. 0.09 [0.01, 0.16]; medial obia: −0.47 [−0.55, −0.40] vs. −0.37 [−0.44 to −0.30]; lateral obia: −0.54 [−0.61, −0.46] vs. −0.39 [−0.46, −0.30]).[22]

### Statistical Analyses

#### Modeling the Influence of BMI and Alignment on the Rate of Cartilage Thinning

To evaluate the independent and interactive effects of BMI and lower limb alignment on changes in longitudinal cartilage thickness, we fit linear mixed-effects models for each cartilage region: weight-bearing medial and lateral femur, medial and lateral tibia. For each region, cartilage thickness was modeled as a function of time, BMI, and HKA alignment. To determine if BMI and lower limb alignment compounded one another to increase the rate of cartilage loss, we initially specified a three-way interaction (time x BMI x HKA alignment) with all two-way interactions and main effects included. If the three-way interaction was not significant (p > 0.05), the model was reduced and refit with only two-way interactions. All models were adjusted for age, sex, and baseline Kellgren-Lawrence grade. All continuous predictors (BMI, HKA, age) were z-score normalized (i.e., divided by their standard deviation [SD]) prior to model fitting. For interpretability, model-derived coefficients for the z-score normalized predictors (BMI, HKA angle, and their interactions with time) were subsequently transformed back into physically interpretable units (i.e., mm of cartilage thickness change per year per kg/m^2^ or per degree of alignment). To account for correlation within subjects and between limbs, we included a subject-level random intercept, a limb-within-subject random intercept, and a limb-within-subject random slope for time.

#### Modeling the Effect of BMI and Alignment on Risk of Total Knee Replacement

To evaluate how BMI and HKA alignment influence the risk of progressing to TKR, we employed a mixed-effects logistic regression model, with TKR occurrence as the binary outcome. Because exploratory analysis of binned data revealed that the risk of TKR was roughly symmetric about the mean, for both varus and valgus malalignments, HKA values were transformed into an absolute deviation from the mean HKA value (−1.2°). This transformation isolates degree of malalignment as a singular risk factor, regardless of direction (varus or valgus). This absolute deviation, along with BMI and age, were z-score normalized to facilitate model convergence.

The primary model included a two-way interaction between BMI and HKA deviation, along with their main effects, while adjusting for baseline age, sex, and KL grade. A subject-level random intercept accounted for the correlation between limbs within each individual. If the two-way interaction was not significant, the model was reduced and refit without the interaction term to estimate the independent effects. For clinical interpretability, coefficients were transformed back into physical units and reported as adjusted Odds Ratios (OR).

Data generated in this study, including quantitative cartilage thickness outcomes, statistical analysis scripts, and model outputs, are publicly available at https://github.com/kenziew/oai_hka_bmi.

## RESULTS

### Dataset

After applying inclusion and exclusion criteria, the final sample consisted of 5,832 limbs from 3,016 participants for a total of 23,699 MRI examinations. Of these, 2,816 subjects (93.4%) had both limbs included, while 200 subjects (6.6%) had only one limb included. Participants had a mean age of 62.6 ± 9.1 years (range: 46-82), mean BMI of 28.5 ± 4.7 kg/m^2^ (range: 16.6-48.9) and mean HKA angle of −1.2° ± 3.3° (range: −16.7° to 18.9°). A total of 299 limbs (5.1%) progressed to TKR during follow-up. Demographic and clinical characteristics are summarized in Table 1.

To characterize the distribution of BMI and lower limb alignment in the cohort, we identified a radiographically healthy subgroup of 1,528 limbs from 1,040 subjects that maintained KL grade 0 throughout all follow-up visits. This subgroup had a mean age of 61.4 ± 9.2 years, mean BMI of 27.0 ± 4.3 kg/m^2^, mean HKA of −1.0° ± 2.6°, and was 52.8% female. These categories are descriptive only and were not used in any statistical analyses. Alignment was categorized relative to this subgroup’s HKA distribution: neutral within ±1 SD of this mean (−3.6° to 1.6°), mild as 1-2 SD, moderate as 2-3 SD, and severe as >3 SD. BMI categories followed standard World Health Organization classifications. The cross-tabulation of these groups is shown in Table 1.

### Influence of BMI and Lower Limb Alignment on the Rate of Cartilage Thinning

**Figure 1.**
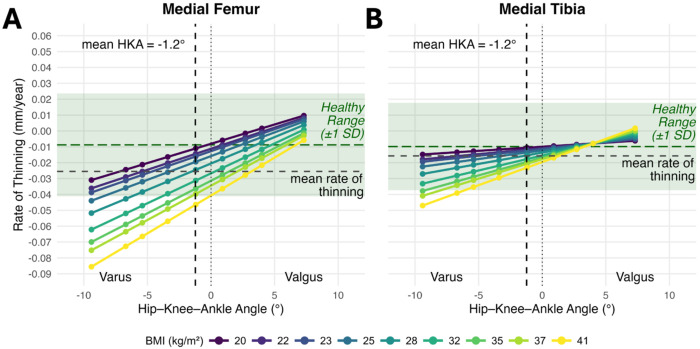
Predicted Annual Rate of Cartilage Thinning in the Medial Compartments. Predicted rate of thinning (mm/year) is shown as a function of Hip-Knee-Ankle (HKA) angle and Body Mass Index (BMI). A significant three-way interaction was observed between time, BMI, and HKA angle (medial femur: p = 0.011; medial tibia: p < 0.001), demonstrating a compounding effect where higher BMI and greater varus alignment increases the rate of medial cartilage thinning beyond their simple additive risk. Solid lines represent HKA angles at the 1st, 5th, 10th, 25th, 50th, 75th, 90th, 95th, and 99th percentiles (−9.4° to 7.3°), plotted across the same percentile range of BMI (20 to 41 kg/m^2^). Negative HKA values indicate a varus alignment, while positive indicate valgus. The mean HKA angle for the cohort was −1.2° varus (black vertical dashed lines) and the mean thinning rate for the whole cohort was 0.026 mm/year for the medial femur and 0.016 for the medial tibia (horizontal black dashed lines). The green horizontal dashed lines and green shaded areas indicate the thinning rate ±1 standard deviation (SD) of the radiographically healthy subgroup of 1,528 limbs from 1,040 subjects that maintained KL grade 0 throughout all follow-up visits (mean HKA = −1.0° ± 2.6°).

**Figure 2.**
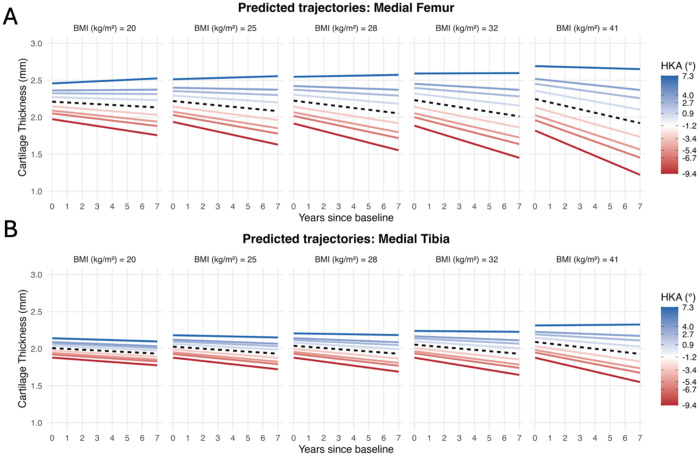
Predicted Longitudinal Cartilage Thickness Trajectories for the Medial Femur (A) and Medial Tibia (B). Panels represent BMI at the 1^st^, 25^th^, 50^th^, 75^th^, and 99^th^ percentiles (20 to 41 kg/m^2^), with colored lines within each panel representing the observed HKA angles at the 1^st^, 5^th^, 10^th^, 25^th^, 50^th^, 75^th^, 90^th^, 95^th^, and 99^th^ percentiles (−9.4°to 7.3°). The black dashed line represents the trajectory for a participant at the cohort mean HKA angle (−1.2°). While divergence between varus (red) and valgus (blue) alignment is present at all BMIs, this gap progressively widens as BMI increases, demonstrating the multiplicative effect of BMI and varus alignment on medial cartilage thinning.

Female sex and older age were both significantly associated with thinner baseline cartilage across all regions (p < 0.001, Supplemental Tables 3–4). At the cohort-average BMI and alignment, the rate of cartilage thinning for the medial femur was 0.026 mm/year (1.34% of mean baseline thickness). There was a significant three-way interaction between time, BMI, and HKA alignment in the medial femur (β = 0.002 mm/year for each 1-SD BMI x 1-SD HKA increase, p = 0.011), indicating that BMI and varus alignment interact to modify the longitudinal rate of cartilage loss (Figure 1A/2A, Supplemental Table 3). To visualize the longitudinal impact of the interaction, we modeled the cartilage thickness trajectories over 7 years, which illustrate how the combination of higher BMI and varus alignment produces greater thinning than either factor alone (Figure 2A). The individual effects of BMI and varus alignment scale linearly with their respective deviations from the mean: each +1 kg/m^2^ above average increases the rate of thinning by 6.6%, and each +1° of varus by 13.4%. The interaction term, however, scales as the product of both deviations (0.44% × ΔBMI × Δvarus). At small increases (+1 kg/m^2^ and +1 ° of varus), the interaction contributes a negligible 0.44%. But at deviations of +10 kg/m^2^ and +10 ° of varus, the interaction alone contributes 43.9%, and when combined with the individual BMI (65.9%) and alignment (133.7%) effects, yields 65.9% + 133.7% + 43.9% = 243.5% faster thinning (Table 2). This multiplicative scaling means the interaction becomes increasingly dominant the further both factors deviate from the mean.

A similar pattern was observed in the medial tibia. At the average BMI and alignment, the rate of cartilage thinning was 0.016 mm/year (0.84% of mean baseline thickness; Figure 1B). The time x BMI x HKA alignment interaction was again significant (β = 0.002 mm/year for each 1-SD BMI x 1-SD HKA increase, p < 0.001; Figure 1B/2B, Supplemental Table 3). The individual effects of BMI and varus alignment were smaller than in the medial femur. For each +1 kg/m^2^ above average, the rate of thinning increased by 3.8%, and for each +1° of varus the rate of thinning increased by 9.6%. The interaction followed the same multiplicative pattern as the medial femur, rising from 0.73% faster thinning at +1 kg/m^2^ and +1 ° varus to 73.2% at +10 kg/m^2^ and +10° varus. The combined 207.1% increase in thinning rate at +10 kg/m^2^ and +10° varus is comprised of 38.0% due to BMI, 95.9% due to malalignment, and 73.2% due to their interaction (Table 2), mirroring the disproportionate compounding seen in the medial femur at greater deviations from the mean.

In contrast, neither lateral compartment showed a significant time x BMI x HKA alignment interaction (lateral femur p = 0.727; lateral tibia p = 0.436), so these regions were modeled using two-way interactions only (time x BMI and time x HKA). At the cohort-average BMI and alignment, thinning rates were 0.005 mm/year (0.26% of mean baseline thickness) in the lateral femur and 0.034 mm/year (1.46% of mean baseline thickness) in the lateral tibia. In the lateral femur, both BMI (β = −0.001, p = 0.002) and alignment (β = −0.002, p < 0.001) independently increased the rate of cartilage thinning. Each +1 kg/m^2^ increased the thinning rate by 5.5%, and each +1° of valgus increased it by 13.8%. The lateral tibia showed a similar but more modest pattern, with each +1 kg/m^2^ increasing the rate of thinning by 0.6% (β = −0.001, p = 0.042) and each +1° of valgus increasing the rate of thinning by 4.2% (β = −0.005, p < 0.001; Supplemental Table 4).

### Influence of BMI and Lower Limb Alignment on the Risk of Total Knee Replacement

**Figure 3.**
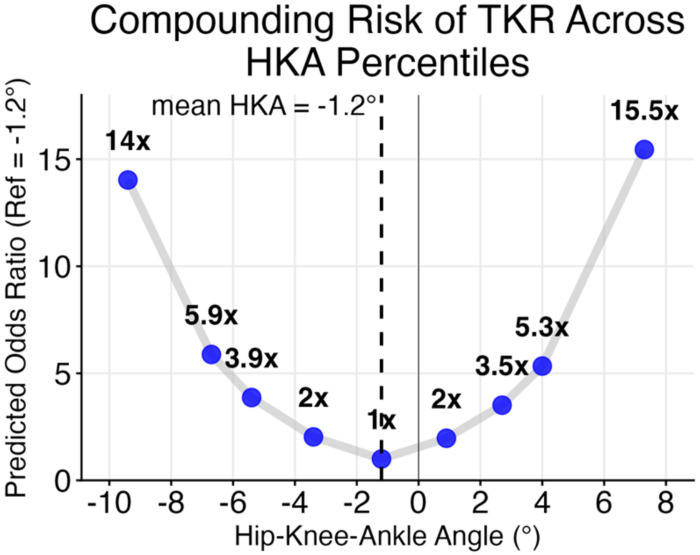
Relative Increase in Risk of Total Knee Replacement by Hip-Knee-Ankle (HKA) Deviation from the Mean HKA Angle. Odds ratios were derived from a mixed-effects logistic regression model adjusted for age, sex, BMI, and baseline KL grade. Blue dots represent HKA angles at the 1^st^, 5^th^, 10^th^, 25^th^, 50^th^, 75^th^, 90^th^, 95^th^, and 99^th^ percentiles (−9.4°to 7.3°). The exponential curve demonstrates a compounding 38% increase in risk of TKR for every 1° of malalignment (OR = 1.38), independent of radiographic OA severity.

There was no statistically significant interaction between BMI and HKA deviation on the risk of TKR (β = −0.123, p = 0.478). In the reduced model, HKA deviation was a significant independent predictor of TKR (β = 0.693, p < 0.001; Supplemental Table 7). The adjusted odds of TKR exponentially increase (1.38x) per 1° greater deviation in alignment. This compounding risk profile means a 5° deviation corresponded to a five-fold increase in the odds of TKR compared with mean HKA alignment (Figure 3). In contrast, BMI did not statistically associate with TKR risk (β = −0.068, p = 0.832).

## DISCUSSION

In this large-scale longitudinal study of 3,016 individuals followed for up to 7 years, we demonstrate that BMI and lower limb alignment influence structural KOA progression through pathways that are fundamentally compartment-specific. In the medial compartment, BMI and varus alignment interacted, such that their combined effect on the rate of cartilage thinning exceeded the sum of their independent contributions. In the lateral compartment, BMI and valgus alignment were independently associated with faster cartilage thinning. Finally, the risk of TKR was strongly and exponentially driven by the degree of malalignment, but not BMI. Together, these findings demonstrate that the relationships between BMI, lower limb alignment, and structural KOA progression are compartment-specific, and identify a discrete high-risk phenotype for medial OA progression.

The multiplicative interaction observed in the medial compartment is consistent with the mechanical framework in which BMI and lower limb alignment function as compounding determinants of joint loading. Specifically, beyond the independent effects of BMI and alignment, which are themselves clinically meaningful, the combined interaction effect grows disproportionately as each factor increases. For example, at +2 units in both BMI (kg/m^2^) and alignment (° varus), the interaction alone increases the rate of medial femur cartilage thinning by 1.8%, at +5 units each it increases the rate by 11.0%, and at +10 units each by 43.9% (Table 2). Similar patterns were observed in the medial tibia, where the interaction alone corresponds to 2.9% faster thinning at +2 units, 18.3% faster thinning at +5 units, and 73.2% at +10 units. These compounding results demonstrate that individuals with both high BMI and varus alignment have a disproportionately elevated risk of structural KOA progression in the medial compartment that cannot be captured by considering either factor in isolation. Across prior studies, alignment has been shown to modulate the effects of BMI on radiographic KOA,[6, 12, 13, 23] yet none characterized a multiplicative interaction. The most direct comparison to the current study is by Moyer et al. who leveraged quantitative MRI from the OAI but used the FTA angle obtained from knee only radiographs as a surrogate for full-limb HKA and did not find a multiplicative interaction.[11] The inconsistencies of prior work likely reflect shared methodological constraints including reliance on JSN or FTA-based alignment surrogates, dichotomized groupings of BMI or alignment, and sample sizes too small to detect interactions.[5–9, 11–13, 17, 24] The present study overcomes these limitations by leveraging a large-scale cohort and direct quantitative measurements of cartilage thickness and lower limb alignment, providing evidence that BMI and varus alignment interact to increase the rate of medial compartment cartilage thinning.

In contrast to the medial compartment, BMI and valgus alignment did not interact and showed only independent effects on lateral cartilage thinning. This absence of compounding does not contradict a mechanical basis for lateral OA progression but suggests that the lateral compartment may respond differently to load. One plausible explanation is that during gait, the medial side is loaded to a greater extent, accepting approximately 66% of total joint load.[25] Because the medial compartment already serves as the primary load-bearing surface, it is uniquely susceptible to factors that compound along this pathway: BMI increases the total magnitude of load while varus alignment increases the proportion of load carried by the medial compartment,[24] together exceeding the load-bearing capacity of the medial cartilage to a degree that neither factor produces alone. This biomechanical framework is consistent with gait studies linking the knee adduction moment (KAM), a widely used surrogate of medial relative to lateral compartment loading, to medial cartilage loss[26, 27] and with findings that KAM interacts with BMI to predict medial tibial cartilage loss over 2.5 years.[28] Because this medial loading bias even persists in valgus knees,[29] the lateral compartment does not fully assume the role of primary load-bearer, which may explain why BMI and valgus alignment contribute independently rather than multiplicatively to lateral cartilage thinning.

The compartment-specific asymmetry between medial and lateral compartment findings raises a broader question about what constitutes a “neutral” alignment. In our TKR analysis, any deviation from the cohort mean (−1.2°) was associated with increased risk of TKR, suggesting that the mechanically “safe” alignment for the knee may not be 0° but slightly varus. Supporting this interpretation, the average alignment of limbs that remained radiographically healthy throughout follow-up (KL grade 0 at all timepoints) had an average HKA angle of −1.0°. Together these two observations suggest that a slight varus position reflects a load distribution that preserves structural integrity across both compartments over time. Consequently, varus and valgus deviations of equal magnitude do not impose equivalent compartment-specific risk, and alignment effects on cartilage should not be assumed to operate symmetrically in both directions from 0°. The identification of a slight varus position as the mechanically favorable reference point may also have implications for surgical planning. A recent randomized controlled trial demonstrated that medial opening wedge high tibial osteotomy (HTO) slowed medial compartment cartilage loss in patients with varus-aligned KOA, demonstrating that mechanical realignment can modify structural disease trajectories.[30] Our findings identify the phenotype, elevated BMI combined with varus malalignment, most likely to benefit from such interventions.

Despite the compartment-specific differences observed for rates of cartilage thinning, TKR risk showed a consistent and exponential relationship with the degree of malalignment regardless of direction. The adjusted odds of TKR were 1.38x per 1° of HKA deviation from average alignment, corresponding to an approximately five-fold increase in TKR odds at 5° of malalignment (approximately 6° varus, 4° valgus; Figure 3). In contrast, BMI was not a predictor of TKR risk. This dissociation between BMI’s role in increasing the rate of cartilage thinning and its lack of association with TKR risk appears counterintuitive but may reflect systematic biases inherent to the surgical selection process.[31] Many centers apply upper BMI thresholds ranging from 35-55 kg/m^2^ for TKR eligibility due to concerns about perioperative complications and inferior outcomes in patients with obesity,[31] which would suppress BMI and TKR associations. Furthermore, TKR also serves to correct for varus or valgus malalignment,[32, 33] and may favor patients with deformity, augmenting the alignment and TKR association observed with the current cohort. Further work is needed to distinguish the influence of surgical selection criteria from underlying structural disease progression in determining which patients ultimately progress to TKR. These findings also have implications for disease-modifying OA drug (DMOAD) trial endpoint development. Several proposed intermediate endpoints for clinical trials, including end-stage KOA (esKOA) and the Composite Knee Osteoarthritis Symptom Outcome (CKOASO), were developed or validated against subsequent TKR as a measure of clinical relevance.[34–36] If TKR is systematically less likely in high-BMI patients regardless of structural severity, these endpoints may inherit the same surgical selection bias, potentially underweighting BMI as a structural risk factor and miscalibrating progression thresholds for patients at substantial risk of structural deterioration. BMI and alignment stratification should therefore be considered essential design features in both trial enrollment and endpoint validation.

Taken together, our findings for rates of compartment-specific cartilage thinning and TKR risk have implications for personalized risk modification in KOA. First, high BMI poses substantial risk to cartilage structural integrity across the full spectrum of alignment values, where even modest increases in BMI were associated with faster thinning in both compartments. In practice, this supports the use of weight-loss[37] strategies, including lifestyle interventions targeting diet and exercise[38] and, where appropriate, Glucagon-like peptide-1 (GLP-1) based pharmacotherapies,[39, 40] as potential structural risk-modifying approaches for KOA. Second, lower limb malalignment is an independent and dominant driver of both compartment-specific cartilage loss and TKR risk, even at normal BMI. Individuals with varus or valgus malalignment may benefit from conservative approaches such as knee bracing to reduce pain and improve symptoms.[41–43] In patients with substantial malalignment or failure of conservative management, particularly younger or more active individuals who are not yet candidates for arthroplasty, surgical realignment procedures such as HTO may be appropriate.[44, 45] Third, individuals with both elevated BMI and pronounced malalignment represent the most at risk group and therefore, combined approaches targeting both BMI and alignment are likely required. Notably, approximately 1 in 7 limbs in our cohort presented with both obesity and malalignment (HKA outside the neutral range −3.6° to 1.6°), suggesting this high-risk phenotype is common (Table 1). Dedicated clinical trials evaluating whether interventions that modify BMI, alignment, or both, slow the rate of cartilage loss and reduce the risk of TKR are needed to translate these observational findings into evidence-based treatment strategies.

While this study has several notable strengths, there are several limitations that warrant consideration. The HKA angle is a frontal-plane metric that does not capture the full complexity of lower limb alignment, including the origin of deformity (femur vs. tibia), rotational alignment, or sagittal plane deformities. Furthermore, HKA measurements were obtained at a single baseline timepoint and reflect static alignment only; the dynamics of malalignment during gait further modulate compartment-specific loading and should be considered in future analyses. Although BMI was used as the primary exposure to reflect standard clinical practice, it imperfectly captures mechanical joint loading. Substituting body mass for BMI produced consistent results across all primary outcomes (Supplemental Tables 5 and 6). Finally, the associations reported here are observational, evaluation of the effects of intervention-induced changes in BMI and alignment on cartilage loss trajectories and TKR risk is needed to establish whether these risk factors are truly modifiable targets for structural joint preservation. To support reproducibility and future investigation, all quantitative cartilage thickness outcomes, analysis scripts, and model outputs are publicly available at https://github.com/kenziew/oai_hka_bmi.

In summary, this large-scale longitudinal study demonstrates that BMI and lower limb alignment interact multiplicatively to increase the rate of medial compartment cartilage thinning, while exerting independent effects in the lateral compartment, and that TKR risk is driven by the severity of malalignment independent of BMI. These findings identify patients with elevated BMI and pronounced malalignment as a high-risk phenotype for structural progression, and directly implicate both factors as targets for intervention, either in isolation or in combination, for structural joint preservation.

## Supplementary Material

1

## Figures and Tables

**Table 1:** Characteristics of the Study Cohort and Distribution of BMI and Alignment.

	Limbs (n)	Subjects (n)	Age	
**Total Sample**	**5832**	**3016**	**62.6 ± 9.1 years**	
Female		1722 (57 %)	63.5 ± 9.0 years	
Male		1294 (43 %)	62.5 ± 9.3 years	
**Number of Longitudinal Follow-up Visits per Limb/Subject (Total # of MRIs)**				
2	705	293		
3	1028	478		
4	1965	923		
5	1459	864		
6	675	458		
**MRI examinations per Follow-up Visit**				
Baseline	5808			
1 year	4978			
2 years	3509			
3 years	2937			
4 years	1754			
5 years	2424			
6 years	1289			
7 years	1000			
**Descriptive Body Mass Index and Alignment Categories at Baseline Per Limb**				
	Underweight (≤18.5 kg/m^2^)	Healthy (18.5-24.9 kg/m^2^)	Overweight (25.0-29.9 kg/m^2^)	Obese (≥30 kg/m^2^)
Severe Varus < −8.8°	0	15	38	44
Moderate Varus ≥ −8.8° to < −6.2°	0	49	112	125
Mild Varus ≥ −6.2° to < −3.6°	1	234	367	315
Neutral ≥ −3.6° to ≥ 1.6°	13	830	1393	1217
Mild Valgus > 1.6° to ≤ 4.2°	3	203	315	288
Moderate Valgus > 4.2° to ≤ 6.8°	1	44	72	76
Severe Valgus > 6.8°	0	14	34	29

Alignment categories defined relative to a radiographically healthy subgroup (KL grade 0 throughout follow-up; n = 1,040 subjects, 1,528 limbs; mean HKA = −1.0° ± 2.6°). Neutral = ±1 SD (−3.6° to 1.6°); Mild = 1–2 SD; Moderate = 2–3 SD; Severe = >3 SD.

**Table 2.** Compounding Effects of BMI and Alignment on Medial Femoral and Tibial Cartilage Loss. Predicted medial cartilage thinning rates across incremental risk categories compared to the average thinning rate of an individual with mean BMI (28.5 kg/m^2^) and mean HKA angle (−1.2° varus). Calculations utilize cohort-specific standard deviations (SD_BMI_=4.7; SD_HKA_=3.3) to translate standardized interaction model coefficients into clinically interpretable units. All rates and percentages were calculated from the full model including intercept, main effects, and the interaction of BMI and alignment. The interaction effect quantifies the compounding risk beyond the additive effects of each individual factor. Each unit increase corresponds to a simultaneous +1 kg/m^2^ increase in BMI and +1° increase in varus alignment relative to the reference individual.

	Increase level	BMI Effect (%)	Varus Effect (%)	Interaction Effect (%)	Total Increase (%)	Thinning Rate (mm/year)
Reference	-	-	-	-	-	−0.026
Medial Femur	+1 Unit	6.6 %	13.4 %	0.4 %	20.4 %	−0.031
	+2 Unit	13.2 %	26.7 %	1.8 %	41.7 %	−0.036
	+5 Unit	33.0 %	66.9 %	11.0%	110.9 %	−0.054
	+10 Unit	65.9 %	133.7 %	43.9 %	243.5 %	−0.088
Reference	-	-	-	-	-	−0.016
Medial Tibia	+1 Unit	3.8 %	9.6 %	0.7 %	14.1%	−0.018
	+2 Unit	7.6 %	19.2 %	2.9 %	29.7 %	−0.020
	+5 Unit	19.0 %	47.9 %	18.3 %	85.2 %	−0.029
	+10 Unit	38.0 %	95.9 %	73.2 %	207.1 %	−0.048

## References

[1] Collaborators, G.B.D.O., Global, regional, and national burden of osteoarthritis, 1990-2020 and projections to 2050: a systematic analysis for the Global Burden of Disease Study 2021. Lancet Rheumatol, 2023. 5(9): p. e508–e522. 37675071 10.1016/S2665-9913(23)00163-7 PMC10477960

[2] Deveza L.A., , Knee osteoarthritis phenotypes and their relevance for outcomes: a systematic review. Osteoarthritis Cartilage, 2017. 25(12): p. 1926–1941. 28847624 10.1016/j.joca.2017.08.009

[3] Karsdal M.A., , Disease-modifying treatments for osteoarthritis (DMOADs) of the knee and hip: lessons learned from failures and opportunities for the future. Osteoarthritis Cartilage, 2016. 24(12): p. 2013–2021. 27492463 10.1016/j.joca.2016.07.017

[4] Hunter D.J., , Biomarkers for osteoarthritis: current position and steps towards further validation. Best Pract Res Clin Rheumatol, 2014. 28(1): p. 61–71. 24792945 10.1016/j.berh.2014.01.007 PMC4010869

[5] Reijman M., , Body mass index associated with onset and progression of osteoarthritis of the knee but not of the hip: the Rotterdam Study. Ann Rheum Dis, 2007. 66(2): p. 158–62. 16837490 10.1136/ard.2006.053538 PMC1798486

[6] Yusuf E., , Body mass index and alignment and their interaction as risk factors for progression of knees with radiographic signs of osteoarthritis. Osteoarthritis Cartilage, 2011. 19(9): p. 1117–22. 21722745 10.1016/j.joca.2011.06.001

[7] Felson D.T., , Valgus malalignment is a risk factor for lateral knee osteoarthritis incidence and progression: findings from the Multicenter Osteoarthritis Study and the Osteoarthritis Initiative. Arthritis Rheum, 2013. 65(2): p. 355–62. 23203672 10.1002/art.37726 PMC3558618

[8] Sharma L., , Varus and valgus alignment and incident and progressive knee osteoarthritis. Ann Rheum Dis, 2010. 69(11): p. 1940–5. 20511608 10.1136/ard.2010.129742 PMC2994600

[9] Sharma L., , The role of knee alignment in disease progression and functional decline in knee osteoarthritis. JAMA, 2001. 286(2): p. 188–95. 11448282 10.1001/jama.286.2.188

[10] Felson D.T., Osteoarthritis as a disease of mechanics. Osteoarthritis Cartilage, 2013. 21(1): p. 10–5. 23041436 10.1016/j.joca.2012.09.012 PMC3538894

[11] Moyer R., Wirth W., and Eckstein F., Longitudinal Changes in Magnetic Resonance Imaging-Based Measures of Femorotibial Cartilage Thickness as a Function of Alignment and Obesity: Data From the Osteoarthritis Initiative. Arthritis Care Res (Hoboken), 2017. 69(7): p. 959–965. 27696763 10.1002/acr.23096 PMC5585678

[12] Sharma L., , The mechanism of the effect of obesity in knee osteoarthritis: the mediating role of malalignment. Arthritis Rheum, 2000. 43(3): p. 568–75. 10728750 10.1002/1529-0131(200003)43:3<568::AID-ANR13>3.0.CO;2-E

[13] Niu J., , Is obesity a risk factor for progressive radiographic knee osteoarthritis? Arthritis Rheum, 2009. 61(3): p. 329–35. 19248122 10.1002/art.24337 PMC2802836

[14] Amin S., , The relationship between cartilage loss on magnetic resonance imaging and radiographic progression in men and women with knee osteoarthritis. Arthritis Rheum, 2005. 52(10): p. 3152–9. 16200595 10.1002/art.21296

[15] Guermazi A., , Why radiography should no longer be considered a surrogate outcome measure for longitudinal assessment of cartilage in knee osteoarthritis. Arthritis Res Ther, 2011. 13(6): p. 247. 22136179 10.1186/ar3488 PMC3334624

[16] Sharma L., , Relationship of meniscal damage, meniscal extrusion, malalignment, and joint laxity to subsequent cartilage loss in osteoarthritic knees. Arthritis Rheum, 2008. 58(6): p. 1716–26. 18512777 10.1002/art.23462

[17] Iranpour-Boroujeni T., , A new method to measure anatomic knee alignment for large studies of OA: data from the osteoarthritis initiative. Osteoarthritis Cartilage, 2014. 22(10): p. 1668–74. 25278076 10.1016/j.joca.2014.06.011

[18] Gatti A.A. and Maly M.R., Automatic knee cartilage and bone segmentation using multistage convolutional neural networks: data from the osteoarthritis initiative. MAGMA, 2021. 34(6): p. 859–875. 34101071 10.1007/s10334-021-00934-z

[19] Gatti A.A., , Investigating acute changes in osteoarthritic cartilage by integrating biomechanics and statistical shape models of bone: data from the osteoarthritis initiative. MAGMA, 2022. 35(5): p. 861–873. 35286512 10.1007/s10334-022-01004-8 PMC8919909

[20] Gatti A.A., , ShapeMed-Knee: A Dataset and Neural Shape Model Benchmark for Modeling 3D Femurs. IEEE Trans Med Imaging, 2025. 44(3): p. 1140–1152. 39453794 10.1109/TMI.2024.3485613 PMC11913582

[21] Eckstein F. and Wirth W., Quantitative cartilage imaging in knee osteoarthritis. Arthritis, 2011. 2011: p. 475684. 22046518 10.1155/2011/475684 PMC3200067

[22] White M.S., , Deep Learning Models Show Greater Longitudinal Sensitivity to Cartilage Thickness Change than Manual Segmentations in Osteoarthritis Initiative Foundation for the National Institutes of Health Cohort, in Orthopaedic Research Society Annual Meeting. 2026, Orthopaedic Research Society: United States.

[23] Felson D.T., , The effect of body weight on progression of knee osteoarthritis is dependent on alignment. Arthritis Rheum, 2004. 50(12): p. 3904–9. 15593215 10.1002/art.20726

[24] Messier S.P., , Influences of alignment and obesity on knee joint loading in osteoarthritic gait. Osteoarthritis Cartilage, 2014. 22(7): p. 912–7. 24857973 10.1016/j.joca.2014.05.013 PMC4108726

[25] Kumar D., Manal K.T., and Rudolph K.S., Knee joint loading during gait in healthy controls and individuals with knee osteoarthritis. Osteoarthritis Cartilage, 2013. 21(2): p. 298–305. 23182814 10.1016/j.joca.2012.11.008 PMC3804122

[26] Bennell K.L., , Higher dynamic medial knee load predicts greater cartilage loss over 12 months in medial knee osteoarthritis. Ann Rheum Dis, 2011. 70(10): p. 1770–4. 21742637 10.1136/ard.2010.147082

[27] Chehab E.F., , Baseline knee adduction and flexion moments during walking are both associated with 5 year cartilage changes in patients with medial knee osteoarthritis. Osteoarthritis Cartilage, 2014. 22(11): p. 1833–9. 25211281 10.1016/j.joca.2014.08.009 PMC4369510

[28] Brisson N.M., , Baseline knee adduction moment interacts with body mass index to predict loss of medial tibial cartilage volume over 2.5 years in knee Osteoarthritis. J Orthop Res, 2017. 35(11): p. 2476–2483. 28323351 10.1002/jor.23564

[29] Johnson F., Leitl S., and Waugh W., The distribution of load across the knee. A comparison of static and dynamic measurements. J Bone Joint Surg Br, 1980. 62(3): p. 346–9. 7410467 10.1302/0301-620X.62B3.7410467

[30] Birmingham T.B., , High Tibial Osteotomy for Medial Compartment Knee Osteoarthritis : A Randomized Trial With Parallel Preference Arm. Ann Intern Med, 2025. 178(9): p. 1238–1248. 40720836 10.7326/ANNALS-25-00920

[31] Sherman W.F., , Surgeon Decision-Making for Individuals With Obesity When Indicating Total Joint Arthroplasty. J Arthroplasty, 2021. 36(8): p. 2708–2715 e1. 33865649 10.1016/j.arth.2021.02.078

[32] Lombardi A.V. Jr., Berend K.R., and Ng V.Y., Neutral mechanical alignment: a requirement for successful TKA: affirms. Orthopedics, 2011. 34(9): p. e504–6. 21902145 10.3928/01477447-20110714-40

[33] Abdel M.P., , Coronal alignment in total knee replacement: historical review, contemporary analysis, and future direction. Bone Joint J, 2014. 96-B(7): p. 857–62. 24986936 10.1302/0301-620X.96B7.33946

[34] Driban J.B., , Defining and evaluating a novel outcome measure representing end-stage knee osteoarthritis: data from the Osteoarthritis Initiative. Clin Rheumatol, 2016. 35(10): p. 2523–30. 27184045 10.1007/s10067-016-3299-5 PMC5033669

[35] Driban J.B., , The Prognostic Potential of End-Stage Knee Osteoarthritis and Its Components to Predict Knee Replacement: Data From the Osteoarthritis Initiative. J Rheumatol, 2023. 50(11): p. 1481–1487. 37657799 10.3899/jrheum.2023-0017 PMC10840653

[36] Collins J.E., , Feasibility and predictive value of proposed early endpoints for randomized controlled trials in knee osteoarthritis. Osteoarthritis Cartilage, 2026. 10.1016/j.joca.2026.02.001 41724426

[37] Gersing A.S., , Is Weight Loss Associated with Less Progression of Changes in Knee Articular Cartilage among Obese and Overweight Patients as Assessed with MR Imaging over 48 Months? Data from the Osteoarthritis Initiative. Radiology, 2017. 284(2): p. 508–520. 28463057 10.1148/radiol.2017161005 PMC5548450

[38] Shahid A., , Comparison of weight loss interventions in overweight and obese adults with knee osteoarthritis: A systematic review and network meta-analysis of randomized trials. Osteoarthritis Cartilage, 2025. 33(4): p. 518–529. 39233046 10.1016/j.joca.2024.08.012

[39] Bliddal H., , Once-Weekly Semaglutide in Persons with Obesity and Knee Osteoarthritis. N Engl J Med, 2024. 391(17): p. 1573–1583. 39476339 10.1056/NEJMoa2403664

[40] Cheng J., , Effect of glucagon-like peptide-1 receptor agonists in osteoarthritis: A systematic review of pre-clinical and human studies. Osteoarthr Cartil Open, 2025. 7(1): p. 100567. 39995585 10.1016/j.ocarto.2025.100567 PMC11849622

[41] Alfatafta H., , Effect of using knee valgus brace on pain and activity level over different time intervals among patients with medial knee OA: systematic review. BMC Musculoskelet Disord, 2021. 22(1): p. 687. 34384421 10.1186/s12891-021-04513-0 PMC8362244

[42] Moyer R.F., , Biomechanical effects of valgus knee bracing: a systematic review and meta-analysis. Osteoarthritis Cartilage, 2015. 23(2): p. 178–88. 25447975 10.1016/j.joca.2014.11.018

[43] Starkey S.C., , Does Valgus Bracing Reduce Estimates of Medial Tibial, Medial Femoral, and Patella Cartilage Contact Pressure in Varus Malaligned Medial Knee Osteoarthritis? Clin Orthop Relat Res, 2025. 483(10): p. 1969–1982. 40668179 10.1097/CORR.0000000000003611 PMC12453385

[44] Birmingham T.B., , Medial opening wedge high tibial osteotomy: a prospective cohort study of gait, radiographic, and patient-reported outcomes. Arthritis Rheum, 2009. 61(5): p. 648–57. 19405016 10.1002/art.24466

[45] Diaz C.C., , Outcomes of Distal Femoral Osteotomy for Valgus Malalignment: A Systematic Review and Meta-analysis of Closing Wedge Versus Opening Wedge Techniques. Am J Sports Med, 2023. 51(3): p. 798–811. 35156408 10.1177/03635465211051740

