# Supplementary material for: BMI and Varus Malalignment Compound to Define a High-Risk Phenotype for Compartment-Specific Knee Osteoarthritis Progression
Source: medRxiv. 2026 Apr 17:2026.04.15.26350819. Preprint. [Version 1] doi: 10.64898/2026.04.15.26350819 (PMC13127543; doi:10.64898/2026.04.15.26350819)
Supplement: 1 [file NIHPP2026.04.15.26350819v1-supplement-1.pdf]

medRxiv preprint doi: https://doi.org/10.64898/2026.04.15.26350819; this version posted April 17, 2026. The copyright holder for this preprint (which was not certified by peer review) is the author/funder, who has granted medRxiv a license to display the preprint in perpetuity.
It is made available under a CC-BY 4.0 International license .

Supplemental:

Table 3: Linear Mixed-Effects Model Results for Medial Femur and Medial Tibia Cartilage Thickness.

Medial Femur			
	β	95% CI	P value
**Main Effects**			
Time (years)	−0.026	[−0.027, −0.024]	<0.001
BMI (z-score)	−0.010	[−0.023, 0.002]	0.110
HKA angle (z-score)	0.154	[0.144, 0.164]	<0.001
Age (z-score)	−0.037	[−0.049, −0.025]	<0.001
Sex (Female vs. Male)	−0.450	[−0.475, −0.425]	<0.001
**KL Grade**			
KL 1 (vs. KL 0)	0.032	[0.008, 0.057]	0.010
KL 2 (vs. KL 0)	0.026	[0.003, 0.048]	0.028
KL 3 (vs. KL 0)	−0.258	[−0.286, −0.231]	<0.001
KL 4 (vs. KL 0)	−0.724	[−0.763, −0.684]	<0.001
**Interactions**			
Time x BMI	−0.008	[−0.009, −0.007]	<0.001
Time x HKA angle	0.011	[0.010, 0.013]	<0.001
BMI x HKA angle	0.021	[0.012, 0.031]	<0.001
Time x BMI x HKA angle	0.002	[0.000, 0.003]	0.011
Medial Tibia			
	β	95% CI	P value
**Main Effects**			
Time (years)	−0.016	[−0.017, −0.015]	<0.001
BMI (z-score)	0.012	[0.001, 0.023]	0.031
HKA angle (z-score)	0.078	[0.070, 0.085]	<0.001
Age (z-score)	−0.046	[−0.057, −0.035]	<0.001
Sex (Female vs. Male)	−0.295	[−0.317, −0.272]	<0.001
**KL Grade**			
KL 1 (vs. KL 0)	0.030	[0.013, 0.048]	0.001
KL 2 (vs. KL 0)	0.054	[0.038, 0.071]	<0.001
KL 3 (vs. KL 0)	−0.047	[−0.067, −0.027]	<0.001
KL 4 (vs. KL 0)	−0.332	[−0.360, −0.305]	<0.001
**Interactions**			
Time x BMI	−0.003	[−0.004, −0.002]	<0.001
Time x HKA angle	0.005	[0.004, 0.006]	<0.001
BMI x HKA angle	0.012	[0.005, 0.019]	0.001
Time x BMI x HKA angle	0.002	[0.001, 0.003]	<0.001

medRxiv preprint doi: https://doi.org/10.64898/2026.04.15.26350819; this version posted April 17, 2026. The copyright holder for this preprint (which was not certified by peer review) is the author/funder, who has granted medRxiv a license to display the preprint in perpetuity.
It is made available under a CC-BY 4.0 International license .

Table 4: Linear Mixed-Effects Model Results for Lateral Femur and Tibia Cartilage Thickness.

Lateral Femur			
	β	95% CI	P value
**Main Effects**			
Time (years)	−0.005	[−0.006, −0.004]	<0.001
BMI (z-score)	0.026	[0.015, 0.037]	<0.001
HKA angle (z-score)	−0.031	[−0.039, −0.023]	<0.001
Age (z-score)	−0.014	[−0.025, −0.003]	0.010
Sex (Female vs. Male)	−0.292	[−0.314, −0.270]	<0.001
**KL Grade**			
KL 1 (vs. KL 0)	0.039	[0.019, 0.058]	<0.001
KL 2 (vs. KL 0)	0.082	[0.064, 0.100]	<0.001
KL 3 (vs. KL 0)	0.040	[0.018, 0.062]	<0.001
KL 4 (vs. KL 0)	−0.077	[−0.108, −0.047]	<0.001
**Interactions**			
Time x BMI	−0.001	[−0.002, −0.000]	0.002
Time x HKA angle	−0.002	[−0.003, −0.001]	<0.001
BMI x HKA angle	0.009	[0.002, 0.017]	0.018
Lateral Tibia			
	β	95% CI	P value
**Main Effects**			
Time (years)	−0.034	[−0.035, −0.033]	<0.001
BMI (z-score)	0.019	[0.003, 0.035]	0.019
HKA angle (z-score)	−0.139	[−0.150, −0.128]	<0.001
Age (z-score)	−0.085	[−0.100, −0.069]	<0.001
Sex (Female vs. Male)	−0.373	[−0.406, −0.342]	<0.001
**KL Grade**			
KL 1 (vs. KL 0)	−0.024	[−0.052, 0.003]	0.085
KL 2 (vs. KL 0)	−0.071	[−0.097, −0.045]	<0.001
KL 3 (vs. KL 0)	−0.297	[−0.328, −0.266]	<0.001
KL 4 (vs. KL 0)	−0.661	[−0.705, −0.617]	<0.001
**Interactions**			
Time x BMI	−0.001	[−0.002, −0.000]	0.042
Time x HKA angle	−0.005	[−0.006, −0.004]	<0.001
BMI x HKA angle	0.004	[−0.007, 0.014]	0.503

medRxiv preprint doi: https://doi.org/10.64898/2026.04.15.26350819; this version posted April 17, 2026. The copyright holder for this preprint (which was not certified by peer review) is the author/funder, who has granted medRxiv a license to display the preprint in perpetuity.
It is made available under a CC-BY 4.0 International license .

Table 5: Linear Mixed-Effects Model Results for Medial Femur and Tibia Cartilage Thickness using Body Weight.

Medial Femur			
	β	95% CI	P value
**Main Effects**			
Time (years)	−0.025	[−0.027, −0.024]	<0.001
Weight (z-score)	0.006	[−0.008, 0.020]	0.422
HKA angle (z-score)	0.153	[0.143, 0.163]	<0.001
Age (z-score)	−0.033	[−0.046, −0.021]	<0.001
Sex (Female vs. Male)	−0.435	[−0.463, −0.406]	<0.001
**KL Grade**			
KL 1 (vs. KL 0)	0.030	[0.006, 0.055]	0.015
KL 2 (vs. KL 0)	0.021	[−0.001, 0.044]	0.067
KL 3 (vs. KL 0)	−0.263	[−0.290, −0.236]	<0.001
KL 4 (vs. KL 0)	−0.726	[−0.765, −0.686]	<0.001
**Interactions**			
Time x Weight	−0.008	[−0.009, −0.007]	<0.001
Time x HKA angle	0.011	[0.010, 0.012]	<0.001
Weight x HKA angle	0.028	[0.019, 0.037]	<0.001
Time x Weight x HKA angle	0.002	[0.001, 0.003]	0.002
Medial Tibia			
	β	95% CI	P value
**Main Effects**			
Time (years)	−0.016	[−0.016, −0.015]	<0.001
Weight (z-score)	0.029	[0.017, 0.042]	<0.001
HKA angle (z-score)	0.077	[0.070, 0.084]	<0.001
Age (z-score)	−0.041	[−0.053, −0.030]	<0.001
Sex (Female vs. Male)	−0.266	[−0.291, −0.241]	<0.001
**KL Grade**			
KL 1 (vs. KL 0)	0.029	[0.011, 0.046]	0.001
KL 2 (vs. KL 0)	0.052	[0.035, 0.068]	<0.001
KL 3 (vs. KL 0)	−0.050	[−0.069, −0.030]	<0.001
KL 4 (vs. KL 0)	−0.332	[−0.361, −0.305]	<0.001
**Interactions**			
Time x Weight	−0.003	[−0.004, −0.002]	<0.001
Time x HKA angle	0.005	[0.004, 0.006]	<0.001
Weight x HKA angle	0.018	[0.011, 0.025]	<0.001
Time x Weight x HKA angle	0.002	[0.001, 0.002]	<0.001

medRxiv preprint doi: https://doi.org/10.64898/2026.04.15.26350819; this version posted April 17, 2026. The copyright holder for this preprint (which was not certified by peer review) is the author/funder, who has granted medRxiv a license to display the preprint in perpetuity.
It is made available under a CC-BY 4.0 International license .

Table 6: Linear Mixed-Effects Model Results for Lateral Femur and Tibia Cartilage Thickness Using Body Weight.

Lateral Femur			
	β	95% CI	P value
**Main Effects**			
Time (years)	−0.005	[−0.006, −0.004]	<0.001
Weight (z-score)	0.056	[0.044, 0.069]	<0.001
HKA angle (z-score)	−0.032	[−0.040, −0.024]	<0.001
Age (z-score)	−0.005	[−0.016, 0.006]	0.361
Sex (Female vs. Male)	−0.237	[−0.262, −0.212]	<0.001
**KL Grade**			
KL 1 (vs. KL 0)	0.036	[0.017, 0.055]	<0.001
KL 2 (vs. KL 0)	0.077	[0.059, 0.095]	<0.001
KL 3 (vs. KL 0)	0.032	[0.010, 0.054]	0.004
KL 4 (vs. KL 0)	−0.083	[−0.114, −0.053]	<0.001
**Interactions**			
Time x Weight	−0.004	[−0.005, −0.003]	<0.001
Time x HKA angle	−0.003	[−0.003, −0.002]	<0.001
Weight x HKA angle	0.003	[−0.004, 0.011]	0.414
Lateral Tibia			
	β	95% CI	P value
**Main Effects**			
Time (years)	−0.034	[−0.035, −0.033]	<0.001
Weight (z-score)	0.047	[0.029, 0.065]	<0.001
HKA angle (z-score)	−0.140	[−0.151, −0.129]	<0.001
Age (z-score)	−0.077	[−0.093, −0.060]	<0.001
Sex (Female vs. Male)	−0.328	[−0.364, −0.292]	<0.001
**KL Grade**			
KL 1 (vs. KL 0)	−0.027	[−0.054, 0.001]	0.056
KL 2 (vs. KL 0)	−0.076	[−0.102, −0.050]	<0.001
KL 3 (vs. KL 0)	−0.304	[−0.335, −0.273]	<0.001
KL 4 (vs. KL 0)	−0.667	[−0.711, −0.623]	<0.001
**Interactions**			
Time x Weight	−0.002	[−0.003, −0.001]	<0.001
Time x HKA angle	−0.005	[−0.006, −0.004]	<0.001
Weight x HKA angle	−0.004	[−0.015, 0.007]	0.444

medRxiv preprint doi: https://doi.org/10.64898/2026.04.15.26350819; this version posted April 17, 2026. The copyright holder for this preprint (which was not certified by peer review) is the author/funder, who has granted medRxiv a license to display the preprint in perpetuity.
It is made available under a CC-BY 4.0 International license .

Table 7. Main-Effects Model Results Predicting Total Knee Replacement Risk

	β	95% CI	P value
**Main Effects**			
HKA angle (z-score)	0.693	[0.325, 1.062]	<0.001
BMI (z-score)	−0.068	[−0.695, 0.559]	0.832
Age (z-score)	−0.237	[−0.874, 0.400]	0.465
Sex (Female vs. Male)	1.145	[−0.165, 2.455]	0.087
**KL Grade**			
KL 1 (vs. KL 0)	1.879	[−0.746, 4.503]	0.161
KL 2 (vs. KL 0)	4.014	[1.585, 6.442]	0.001
KL 3 (vs. KL 0)	6.888	[4.503, 9.273]	<0.001
KL 4 (vs. KL 0)	9.650	[7.048, 12.252]	<0.001
